# Up-regulation of RNA Binding Proteins Contributes to Folate Deficiency-Induced Neural Crest Cells Dysfunction

**DOI:** 10.7150/ijbs.33976

**Published:** 2020-01-01

**Authors:** Wenbo Liu, Kang Wang, Xiaoyan Lv, Qian Wang, Xiu Li, Zhigang Yang, Xia Liu, Li Yan, Xin Fu, Ran Xiao

**Affiliations:** Research Center of Plastic Surgery Hospital, Chinese Academy of Medical Sciences and Peking Union Medical College, 33 Ba-Da-Chu Road, Beijing, 100144, People's Republic of China

**Keywords:** Neural crest cells, neural tube defects, folic acid deficiency, RNA binding protein

## Abstract

Folate deficiency has long been associated with the abnormal development of the neural crest cells (NCCs) and neural tube defects (NTDs). RNA binding proteins (RBPs) also play important roles in the normal neural crest development and neural tube formation. Nevertheless, the causative mechanism by which folate status influences human NCCs development and the RBPs functions remains unknown. In this study, we differentiated H9 human embryonic stem cells into neural crest cells (H9-NCCs) and then constructed three folic acid (FA) deficiency (FAD) H9-NCCs models *in vitro*. Decreased viability, impaired migration and promoted apoptosis of H9-NCCs were observed in three FAD H9-NCCs models. In addition, we showed that three RBPs, namely, *hnRNPC*, *LARP6* and* RCAN2,* were up-regulated both in the FAD H9-NCC models *in vitro* and in the FAD mouse model *in vivo*. Knocking down of these three RBPs increased the H9-NCC viability and RCAN2 knockdown further promoted H9-NCC migration under FAD conditions. In normal culture condition, overexpression of RCAN2 and HnRNPC did not affect viabilities and migration of H9-NCCs while overexpression of LARP6 reduced the H9-NCC viability. Our findings demonstrate important regulatory effects of RBPs underlying FAD-induced impaired function of NCCs.

## Introduction

Neural crest cells (NCCs) are highly multipotent cells with migratory capacities; they arise from the dorsal open neural plate during mammalian embryogenesis 1. NCCs are capable of giving rise to a wide variety of derivatives of different tissues, including melanocytes, peripheral neurons and glial, adrenomedullary cells, cardiovascular connective tissues, and much of the bone and cartilage of the face 2-4. Abnormal migration, differentiation, division or survival of NCC leads to organ and tissue dysplasias with highly diverse clinical and pathological features, collectively known as neurocristopathies 5. Most neurocristopathies have a strong genetic basis 6. Recent studies showed that mutations in genes expressed in the cranial neural crest are also associated with neural tube defects (NTDs) 7-9. NTDs are among the most common birth defects in the world with clinical features of anencephaly, craniorachischisis, and spina bifida 10. The overall prevalence of NTDs ranges from 6.9 to 21.9 per 10000 births 11, and it causes enormous clinical, economic and societal costs.

Neurocristopathies and NTDs are both multifactorial disorders that can be induced by both genetic susceptibility and environmental disturbances. For instance, mutations of either SOX10 or PHOX2B disrupt neural crest development and cause Hirschsprung disease in both human 12, 13 and mouse model 6, 14. More than 20 different transcription factor genes give rise to NTDs when mutated in animal models (e.g., *Grhl3* 15, *Zic2* 16, *Twist* 17, *AP2* 18, *Pax3* 19 *etc*.). Environmental intervention, such as folate deficiency, has long been associated with neurocristopathies and NTDs 20. Various studies demonstrate the pathogenic decrease in folate in the periconceptional period and the role of folic acid (FA) in preventing neurocristopathies 21 and NTDs 22, 23. Folates are a family of B9 vitamins that play critical roles in numerous biological processes. Dietary FA enters the cell through three transporters: folate carriers, folate receptors, and proton coupled folate transporters. In adult tissues, the membrane transport of folates is mainly mediated by reduced folate carrier (RFC, SLC19A1), a ubiquitously expressed bidirectional anion channel that has relatively high affinity with reducing folic acid and folic acid analogs such as methotrexate (MTX) 24. MTX belongs to the class of antifolate therapeutic agents which inhibits the enzyme dihydrofolate reductase, thereby depleting the pool of reduced folates and producing a state of effective folate deficiency 25. Once inside the cell, FA undergoes a series of oxidative/reductive transformations into 5,10-methylene tetrahydrofolate (5,10-CH2-THF) and 10-formyl-THF (10-CHO-THF); these compounds provide one carbon unit (OCUs) for purine nucleotide and thymidylate synthesis respectively, which are essential for the *de novo* production of RNA and DNA 26-28. FA also provides a substrate for the methionine cycle to form S-adenosylmethionine 29. SAM mediates the DNA/RNA/protein/lipid methylation, histone modification and affects gene expression and stability 30.

Variations in folate metabolism and transport have been the most intensively studied group of candidate genes for NTDs. The association between methylenetetrahydrofolate reductase (MTHFR) producing 5-Me-THF from 5,10-methylene THF 31 and variant 677C >T has been recognized as a genetic risk factor of NTDs 32. FA deficiency-induced inhibition of the methylation cycle may cause toxic accumulation of homocysteine and is a possible mechanism of FA deficiency-related NTDs 33. Evidence has emerged to suggest that folate receptors are also critical for neural crest development because recent murine "knock-out" and "knock-down" of folate receptors results in a high percentage of folate-responsive neurocristopathies 34, but there is still a need to further explain the molecular mechanism of FA deficiency-induced abnormal gene expression. The gene expression profile of human fibroblast cells grown in folate-deficient medium suggests an alternative mechanism by which reduced folate is impacting cell function and genes linked to critical signaling pathways, including Wnt and Ras (GSE3548). However, we are interested in the gene expression change at the posttranscriptional level in FA deficient cells and find 38 RNA binding proteins (RBPs) significantly upregulated in the NCBI GEO dataset GSE3548.

RBPs are categorized as the posttranscriptional regulators 35 and exert their posttranscriptional functions, including capping, pre-mRNA splicing, mRNA export, mRNA stability modulation and translation regulation 36. RBP defects can cause a spectrum of pathologies and syndromes with a preponderance of published examples among neurological diseases, muscular atrophies, metabolic disorders, and cancer 37. Controlled gene regulation during neural tube development is vital for maintaining the proper development stages of NCCs, and RBPs can affect all aspects of this highly organized process. For instance, DiGeorge critical region 8 (Dgcr8) is a double-stranded RBP that interacts with Drosha and is essential for miRNA biogenesis. Cranial NCCs in *Dgcr8*-null mice undergo apoptosis, decreasing the progenitor pool required for outflow tract remodeling, which finally leads to cardiovascular defects 38. Vg1 RBP, an RBP implicated in RNA localization in oocytes, is required for the migration of cells forming the roof plate of the neural tube and, subsequently, for neural crest migration 39. Cellular nucleic-acid-binding protein (CNBP), a single-stranded nucleic acid binding protein that is able to bind to single-stranded DNA and RNA molecules, is required for neural crest development by regulating the expression of neural crest genes during the early specification process^40^. Hence, there is a high possibility that under FA deficient conditions, RBPs also play roles in NCC development, in which case, posttranscriptional regulation leading to downstream effects needs to be explored.

In this study, we derived human NCCs from the H9 human embryonic stem cells (hESCs) line and established three human NCC FA deprivation (FAD) models *in vitro* by either culturing NCCs in FA-free (FAF) medium, or treating NCCs with MTX or shRNA-mediated silencing of RFC gene in NCCs. RBPs up-regulated in human FAD NCCs were screened out and then were verified in a mouse FAD model *in vivo*. Finally, the influences of 3 RBPs, namely, HnRNPC, LARP6 and RCAN2, on FAD NCCs were investigated.

## Materials and Methods

### Derivation and Culture of H9-NCCs

The H9 hESCs were cultured on plates pre-coated with Matrigel (BD Biosciences, San Jose, CA) and maintained in the mTeSR™1 Complete Kit (Stem Cell Technologies, Vancouver, Canada) medium with daily medium change. When the cells reached 80% confluence, hESCs were detached from the plate with dispase (1 mg/mL, Solarbio, Beijing, China), passaged at a ratio of 1:6 and seeded on a new Matrigel-coated plate. On the 2nd day after passaging, the hESCs were induced to H9-NCCs with NCC differentiation medium as described 41.

### Differentiation of H9-NCCs to mesenchymal stem cells (MSCs)

For MSCs differentiation, H9-NCCs were treated with Accutase (Thermal Fisher, Carlsbad, CA) and then plated on 0.1% gelatin-coated plate with MSC differentiation medium as described 42. MSCs were subsequently induced into osteoblast by culturing in osteogenic differentiation medium (Cyagen, Santa Clara, CA) and stained by Alizarin Red solution (Cyagen) after 2 weeks of culture.

### Neuronal differentiation of H9-NCCs

For peripheral neuron differentiation, H9-NCCs were treated with Accutase and cultured in peripheral neuron medium as described 41.

### Immunofluorescence

Cells cultured on Matrigel-precoated glass coverslips were fixed, permeabilized followed by blocking with PBS containing 5% normal goat serum (Vector Laboratories, Burlingame, CA) and 2% BSA (Sigma) at room temperature. Incubation was performed overnight at 4°C with primary antibodies, including p75NTR (D4B3) Rabbit mAb (CST, 8238, Danvers, MA), SOX9 Rabbit mAb (CST, 82630), SOX10 Rabbit mAb (Abcam, ab155279, Cambridge, UK), AP2α+β Mouse mAb (Abcam, A6/2/2 ), FOXD3 polyAb (Biolegend, 631702, San Diego, CA), CD57 Mouse mAb (Santa Cruz, sc-81633, Dallas, Texas), Pax-6 (AD2.35) (Santa Cruz, sc-53108), Purified Mouse Anti-Oct-3/4 (BD Bioscience, 611203), Sox2 (D9B8N) Rabbit mAb (CST, 23064S), and Peripherin Rabbit polyAb (Abcam, ab4666). The primary antibody incubations were followed with appropriate secondary antibodies: Alexa Fluor 594 goat anti-rabbit IgG (H+L) (Invitrogen, A-11037), Goat Alexa Fluor 488 goat anti-Mouse IgG (H+L) (Invitrogen, A-11029), Alexa Fluor 594 goat anti-mouse IgG (H+L) (Invitrogen, A-11005), Alexa Fluor 488 goat anti-mouse IgG (H+L) (Invitrogen, A-11001). The slides were mounted with mounting medium with DAPI (ZSGBBio, Beijing, China) to localize the nucleus and then imaged with a Leica DM3000 microscope (Leica Microsystems Gmbh, Wetzlar, Germany).

### Flow Cytometry

The proportions of H9-NCCs expressing NCCs surface markers were quantified by flow cytometry analysis using FITC anti-human CD271 (p75NGFR) antibody (BioLegend, 345104) and APC anti-human CD57 (HNK-1) antibody (BioLegend, 359610). MSCs were characterized using StemflowTM human MSC analysis kit (BD) following manufacturer's instructions. The data with 1×10^4^ events per cell sample were generated by FACSAria™ II (BD) and analyzed using Flowjo v7.6.1 software (Tree Star, OR, US).

### Establishment of NCC Culture Models with FA Deprivation

Three human NCC FAD models were established *in vitro* by either culturing NCCs in FA-free (FAF) medium (FAF model), or treating NCCs with MTX (MTX model), or applying shRNA-mediated silencing of RFC gene in NCCs (shRFC model). For the FAF model, DMEM/F12 in NCC differentiation medium was replaced with FA-free RPMI-1640 (Life Technologies, Rockville, MD) and RPMI-1640 (Life Technologies, Rockville, MD) for FAF group and control group respectively 43. For the MTX model, MTX was added (J&K, Beijing, China) to normal NCC differentiation medium at the concentration of 50 nM. For the shRFC model, the *RFC* gene was silenced with shRNA. The hairpin oligonucleotides specific to human *RFC* were cloned into the PLKO.1 vector. The sequences of the shRNAs are listed in Supplementary Table S1.

### Construction of RBP Overexpression vectors

The vectors encoding full-length RBP genes (RCAN2, LARP6 and HnRNPC) were constructed by integrating the open reading frame of RBP genes into pCDH-CMV-MCS-EF1-copGFP (pCDH-GFP, System Biosciences, Palo Alto, CA, USA) individually.

### Virus Production and infection of H9-NCC

For viral packaging, the vectors were transfected into 293T cells together with psPAX2 and pMD2.G (Addgene, Cambridge, MA) using jetPRIME^®^ Transfection Reagent (PolyPlus-Transfection®, Strasbourg, France). The supernatants containing virus were collected, concentrated and titered before infecting H9-NCCs with polybrene (5 ug/ml; Millipore, Burlington, MA). At 72 h after transfection, the cells were harvested for further analysis.

### Real-Time Polymerase Chain Reaction (PCR)

Total RNA was extracted using Trizol reagent (Invitrogen, Grand Island, NY). The total RNA was reversely transcribed using M-MLV reverse transcriptase (Promega, Madison, WI) according to the manufacturer's instructions. The real-time PCR was performed using the Fast SYBR Green Master Kit and Light Cycler 480 system (Roche, Basel, Switzerland) according to the manufacturer's instructions. Primers are listed in the Supplementary Table S2.

### Western Blot

Cellular proteins were harvested in RIPA buffer on ice and stored in -80℃. Protein concentrations were determined using a BCA Protein Assay kit (Beyotime, Beijing, China). Equivalent amounts of protein were separated by electrophoresis on a 10% SDS-PAGE gel; the proteins were then transferred to polyvinylidene fluoride membrane (Millipore) and incubated at 4°C overnight with antibodies against hnRNPM (Abcam ab177957), hnRNPC1+C2 (Abcam, ab10294), TRMT1(Abcam, ab138831), Glyceraldehyde 3-phosphate dehydrogenase/GAPDH (ZSGB-BIO, TA-08), RCAN2 (Proteintech, 12900-1-AP, Illinois, USA), and PPIL4 (Proteintech, 12538-1-AP). Peroxidase-conjugated AffiniPure Goat anti-Rabbit IgG (ZSGB-BIO, ZB-2301, 1/5000) and anti-mouse IgG (ZSGB, ZB-2305, 1/5000) were used and SuperSignal® West Pico Trial Kit (Thermo Fisher Scientific) was applied for protein detection.

### ELISA Analysis for FA level

H9-NCCs were digested and resuspended in PBS at a concentration of 10^7^ cell/mL and then subjected to ultrasonication till the solution was clarified; the supernatant was collected by centrifugation at 1500 g for 10 min at 4℃ and the aliquots were stored in triplicate at -80°C for later use. Mouse venous blood was collected through the eyeball under sterile condition; samples were stored at 4 °C for 2 hours to clot, and the serum was separated via centrifugation at 1000 g for 20 min and then the aliquots were frozen at -70°C until analysis. Folate concentrations were determined by ELISA Kit for Folic Acid (Cloud-Clone Corp, Houston, TX).

### Cell Viability Assay

Cell number was quantified using a Cell Counting Kit-8 assay (Dojindo Laboratories, Tokyo, Japan), according to the manufacturer's instructions. Briefly, approximately 5,000 cells were plated in 48-well plates with 200 μl of NCC differentiation medium. Cell number was measured by absorbance at 450 nm on a PerkinElmer EnSpire^TM^ Multimode Plate Reader. All experiments were repeated three times with triplicates in each experiment.

### Cell Apoptosis Analysis

The H9-NCCs were incubated with the Muse Annexin V Dead Cell Kit (Millipore) following the manufacturer's instructions. The percentages of total apoptotic cells were analyzed on the Muse Cell Analyzer (Millipore) in accordance with the manufacturer's instructions.

### Transwell Migration Assay

After 5 days in different culture conditions, H9-NCCs were treated with Accutase (Life Technologies), resuspended in NCC differentiation medium and then seeded at 1×10^5^ cells/well in the 24-well transwell chamber (Millipore, 8-μm pore size). DMEM/F12+20% FBS was added in the lower compartment. After 24 h, cells remaining on the upper sides of the chamber were removed with cotton swabs. Cells on the lower side of the insert were stained with crystal violet. The number of migrated cells was counted under a microscope in 5 randomly chosen fields, and then the crystal violet was eluted by acetic acid and measured at 570 nm on a PerkinElmer EnSpire^TM^ Multimode Plate Reader. All experiments were repeated in triplicate.

### Animal Model

Animal experiments were approved by the Institutional Animal Care and Use Committee of Plastic Surgery Hospital (Institute). Female C57BL/6J mice at 6-7 weeks old were purchased from Ke Ao Xie Li Ltd. (Beijing, China) and randomly assigned to either the control group fed with a standard AIN93G diet or the FAD group fed with FAD diet lacking FA and with 1% succinyl sulfathiazole (J&K) throughout breeding and gestation until sacrifice at 10.5 dpc 44-46. Female mice fed with their respective diets for 12 weeks were mated with C57BL/6J males. Uteri were dissected, and fetuses were inspected for malformations under a dissecting scope (Leica). The crown-rump length (CRL) was determined using Image J by obtaining the standard sagittal section of embryo and measuring the maximum distance from the top of the embryo to the bottom of the hip. The measurements were averaged three times. For enrichment of neural crest-derived cells, the dorsal neural tube portions of embryos were separated from the ventrolateral tissues under the dissecting binocular microscope, and recorded as dorsal group and ventral group, respectively. The embryo tissues were trimmed and dissolved in Trizol reagent for RNA extraction followed by real-time PCR analysis.

### Statistical Analysis

Statistical significances were determined using a two-tailed Student's t-test and one-way analysis of variance (ANOVA) by GraphPad Prism 6.0 software (GraphPad Software, Inc., San Diego, CA). The level of significance was set at P< 0.05. Data are shown as the mean ± SEM (standard error of the mean).

## Results

### Differentiation and verification of H9-NCCs from H9 hESCs

**Figure 1:**
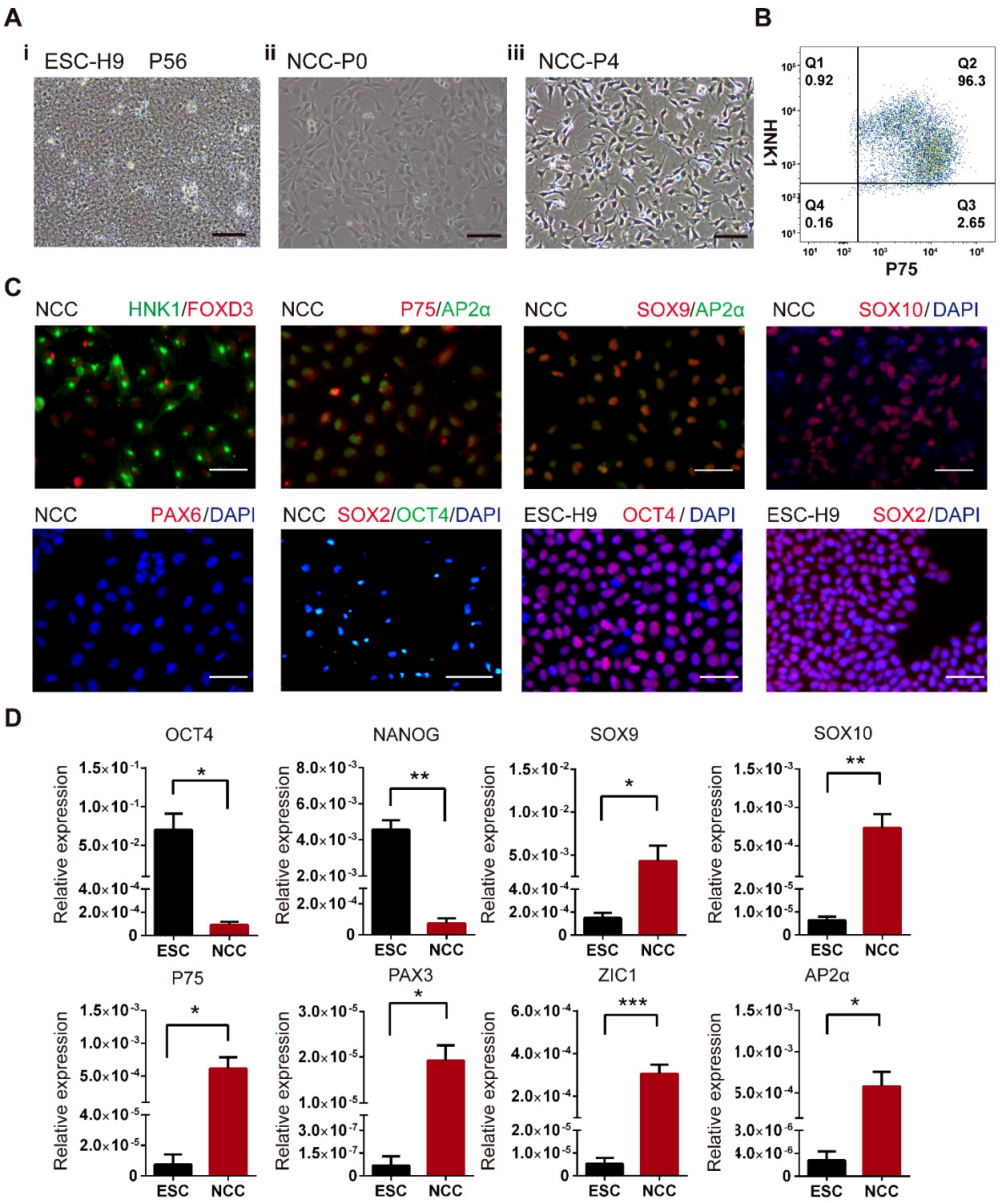
** Derivation and characterization of H9-NCCs differentiated from H9-hESCs.** (A) Phase-contrast images of H9 hESCs at passage 56 (P56) and H9-NCCs and passage 0 (P0) and passage 4 (P4). (B) Flow cytometry analysis of H9-NCCs. (C) Immunocytochemistry analysis showed that H9 hESC-derived H9-NCCs were positive for HNK1, FOXD3, AP2*α*, p75, SOX9, SOX10 and were negative for neural progenitor marker PAX6 and undifferentiated hESCs markers SOX2 and OCT4. (D) Real-time PCR data showed the expression of *OCT4*, *NANOG*,* SOX9*, *SOX10*,* P75*, *PAX3*, *ZIC1* and *AP2α* in H9-NCCs relative to H9 hESCs. Data were shown as the means ± SEM of three independent experiments. Unpaired two-tailed Student's t test. *p < 0.05, **p < 0.01. Scale bars, 100 μm.

The undifferentiated H9 hESCs exhibited the typical compact colonies with distinct borders and a high nucleus-to-cytoplasm ratio with well-defined nuclei (Fig. 1A-i). After 5 days of culture in NCC differentiation medium, a population of stellate-morphology cells migrated away from the cluster of hESCs (Fig. 1A-ii). The cell populations with homologous neural crest morphology were obtained after being cultured in neural crest medium for 2 passages (around 10 days, Fig. 1A-iii). To verify whether these NCC-like cells were authentic NCCs, flow cytometry analysis, immunofluorescent (IF) staining and real-time PCR analysis were used to characterize the expression of NCC markers. More than 95% of the induced cells were double positive for expressions of NCC surface markers HNK1 and P75 (Fig. 1B). IF staining further demonstrated that the induced cells were positive for NCC markers HNK1, P75, FOXD3, AP2α, SOX9, SOX10 but negative for hESCs markers SOX2 and OCT4 and neural progenitor marker PAX6 (Fig. 1C). Moreover, the mRNA levels of the NCC markers *SOX9*, *SOX10*, *P75*, *PAX3*, *ZIC1*, and *AP2α* were significantly higher, but the hESCs markers *OCT4* and *NANOG* were hardly detected in these differentiated cells compared to hESCs (Fig. 1C). The identity of the induced cells was further evaluated by confirming their differentiation potentials to MSCs and peripheral neurons (Fig. S1). Taken together, our results indicated that these differentiated cells possessed NCC characteristics and were termed H9-NCCs.

### FAD Impairs Cell Viability and Migration in H9-NCC models *in vitro*

**Figure 2:**
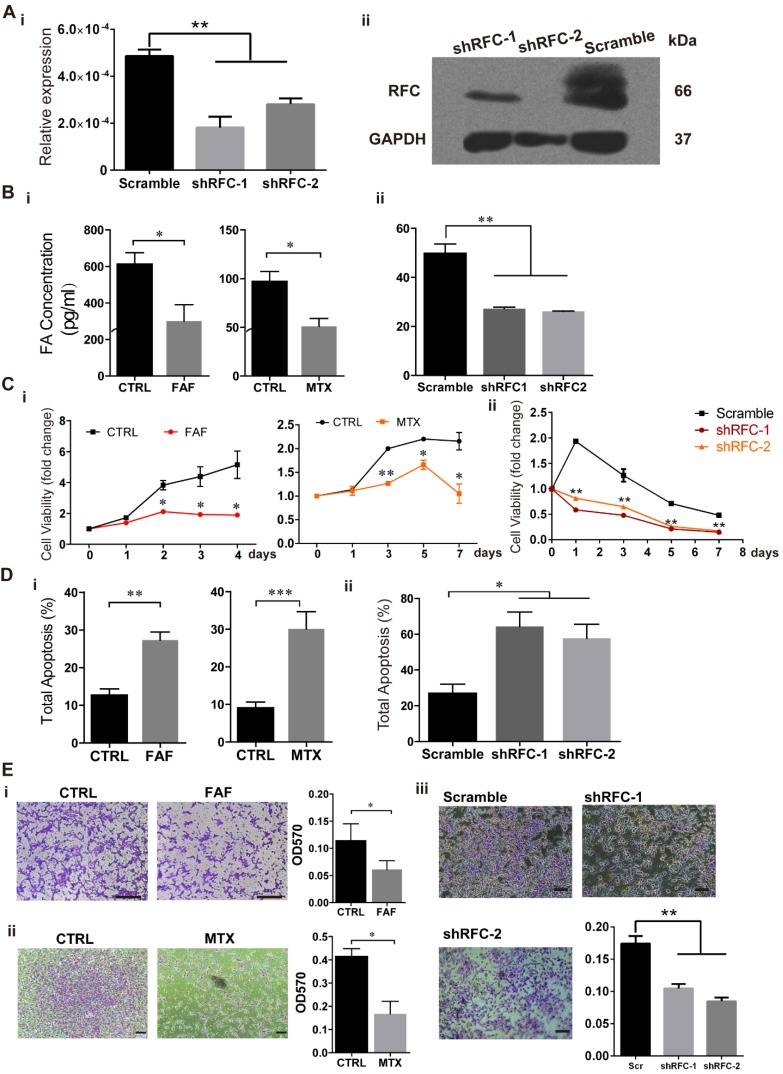
** Folic acid deficiency (FAD) impaired viability and migration and promotes apoptosis of H9-NCC *in vitro*.** (A) Verification of RFC mRNA level by Real-time PCR (i) and RFC protein levels by western blot. (B) H9-NCC FA levels in FAF group and MTX group (i) and in shRFC group; (C) CCK-8 evaluation of H9-NCC viability after FAF/MTX (i) and shRFC treatment; CCK-8 tests showed impaired H9-NCC viability in all groups; (D) Quantification of apoptosis was measured by Annexin-V/7-ADD staining 5 days after treatments to assess the effects of FAF, MTX (i) and shRFC on cell death. * P<0.05, ** P<0.01. Scale bars, 100 μm. (E) Transwell assay results showed that the H9-NCC migration in FAF model, MTX group (i) and shRFC group were all suppressed.

To investigate the effects of FA deprivation on the development of NCCs, we established three FAD H9-NCC models: FAF model, MTX model and shRFC model *in vitro*, which mimicked FA deprivation circumstances of insufficient FA intake, disruption of FA metabolism and genetic defects respectively. For FAF model, H9-NCCs were cultured in either FAF medium (DMEM/F12 in NCC differentiation medium was replaced with FA-free RPMI-1640) or control medium (DMEM/F12 in NCC differentiation medium was replaced with RPMI-1640) for 5 days before subsequent experiments. For MTX model, H9-NCCs in MTX-group were treated with 50 nM MTX while H9-NCCs cultured in NCC differentiation medium were used as control. For shRFC model, the H9-NCCs were infected with virus either carrying shRNAs specific to RFC gene (shRFC group) or scramble shRNA (Scramble group). Three days post-infection, down-regulations of RFC at the mRNA and protein level in shRFC group of H9-NCCs were confirmed by real-time PCR analysis (Fig. 2A-i) and western blotting (Fig. 2A-ii), respectively. ELISAs showed that cellular FA levels of H9-NCCs in the FAF-group (300.54 ± 90.05 pg/mL), the MTX-group (51.16 ± 7.934 pg/mL) and the shRFC-group (26.12 ± 0.1299 pg/mL) were significantly lower than those in the corresponding controls (613.56 ± 61.92; 97.18 ± 10.17; 49.89 ± 3.714 pg/mL, Fig. 2B).

To assess the influence of FAD on H9-NCC viability, the number of viable cells was determined by the CCK8 assay. The viabilities of H9-NCCs in FAF-group and MTX-group were significantly decreased when compared with their corresponding controls after 2 days and 3 days of culture respectively (Fig. 2C-i). For the shRFC-group, the H9-NCC viability was markedly suppressed as early as the first day of culture (Fig. 2C-ii).

The occurrence of cell death may be one reason for the abnormality of NCC development; cell apoptosis was analyzed after Annexin V/7-AAD staining. After treatment for 5 days, there was increased apoptosis in H9-NCCs in both the FAF group (27.33 ± 2.116) and MTX (30.09 ± 4.598) group (Fig 2D-i). The effect of the *RFC* knockdown on H9-NCC apoptosis was even more pronounced (64.34 ± 8.076) (Fig. 2D-ii). The phenotypes also correlated with the cellular FA levels.

The migration of all the three folate-deplete groups were significantly impaired compared to the corresponding controls (Fig. 2E). Collectively, all these data suggest that FA deprivation induces H9-NCC apoptosis and impairs cell viability and migration ability.

### FAD Leads to Up-regulation of RBPs in FAF and shRFC Groups of NCCs *in vitro*

**Figure 3:**
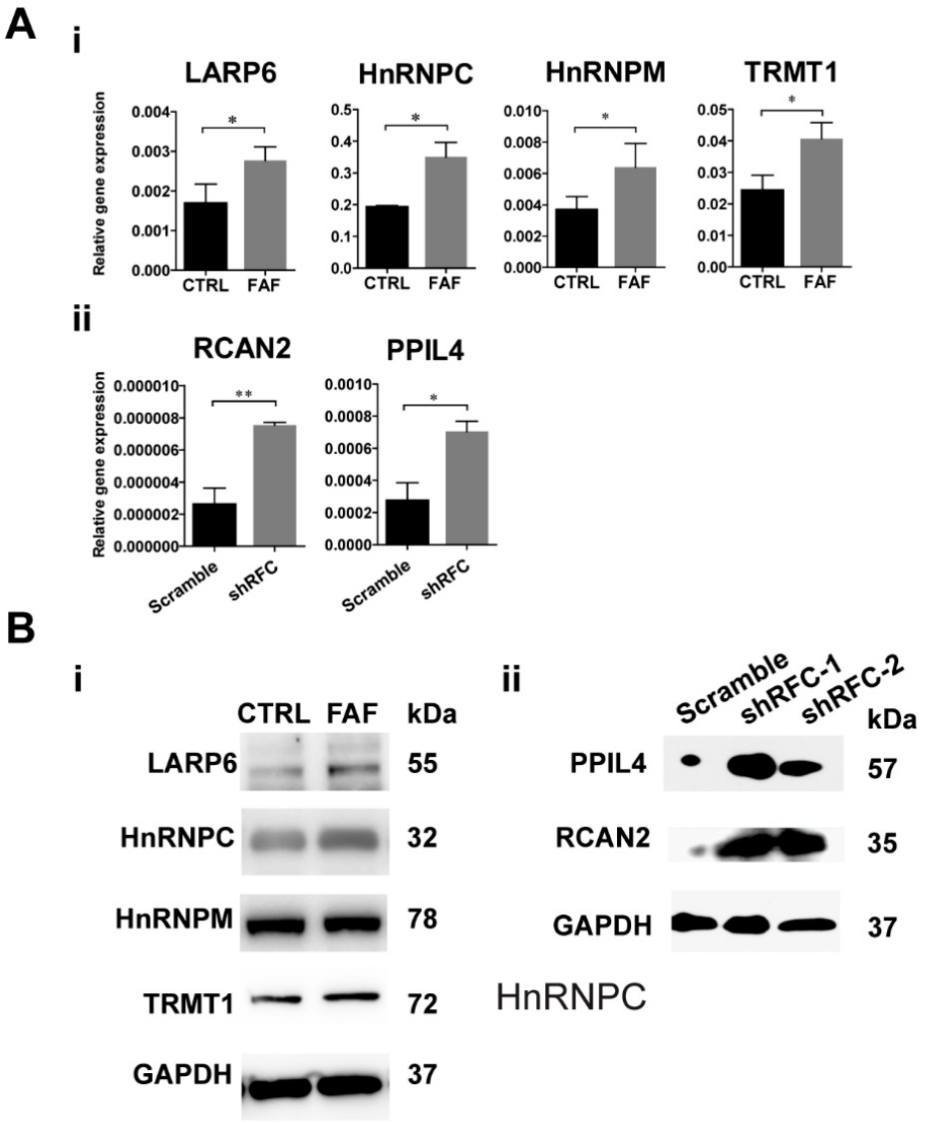
** RBPs were highly expressed in the FAD NCC models *in vitro.*** (A) The validation of GEO dataset GSE3548 data by real-time PCR. The expressions of *HNRNPM*, *HNRNPC*, *LARP6*, and *TRMT1* were significantly up-regulated in FAF-group H9-NCCs compared to control-group H9-NCCs (i). The expressions of *PPIL4* and *RCAN2* were significantly up-regulated in the shRFC-group H9-NCCs compared to Scramble-group H9-NCCs. (B) Western blots showed evidence of up-regulation of LARP6, hnRNPC and TRMT1 proteins in the FAF-group H9-NCCs (i) and PPIL4 and RCAN2 proteins in the shRFC-group H9-NCCs. n≥3. * P<0.05, ** P<0.01. FAD, folic acid deficiency; RFC, reduced folate carrier; hnRNPM, heterogeneous nuclear ribonucleoprotein M; hnRNPC, heterogeneous nuclear ribonucleoprotein C; LARP6, La ribonucleoprotein domain family member 6; TRMT1, tRNA methyltransferase 1; PPIL4, peptidylprolyl isomerase like 4; RCAN2, regulator of calcineurin 2.

The GEO dataset numbered as GSE3548 was chosen to screen out the differently expressed genes under the condition of FAD medium culture. In this microarray assay, approximately 3000 genes were significantly differently expressed, among which were 38 RBPs (Table 1). Changes in transcription levels were verified by real-time PCR analysis and showed that four RBPs (*HNRNPM*, *HNRNPC*, *LARP6*, *TRMT1*) increased significantly in the FAF-group H9-NCCs (Fig. 3A-i), and two RBPs, namely, *PPIL4* and *RCAN2,* were up-regulated significantly in the shRFC-group H9-NCCs (Fig. 3A-ii). However, no overlapping RBP genes were detected in the MTX-group NCCs, probably due to the different FA deprivation circumstances. Western blotting confirmed the increase at the protein level of LARP6, HnRNPC and TRMT1 in FAF- group (Fig. 3B-i) and PPIL4 and RCAN2 in shRFC-group H9-NCCs (Fig. 3B-ii).

### FAD Diet Increases NTDs incidence and Up-regulates RBPs in mouse *in vivo*

**Figure 4:**
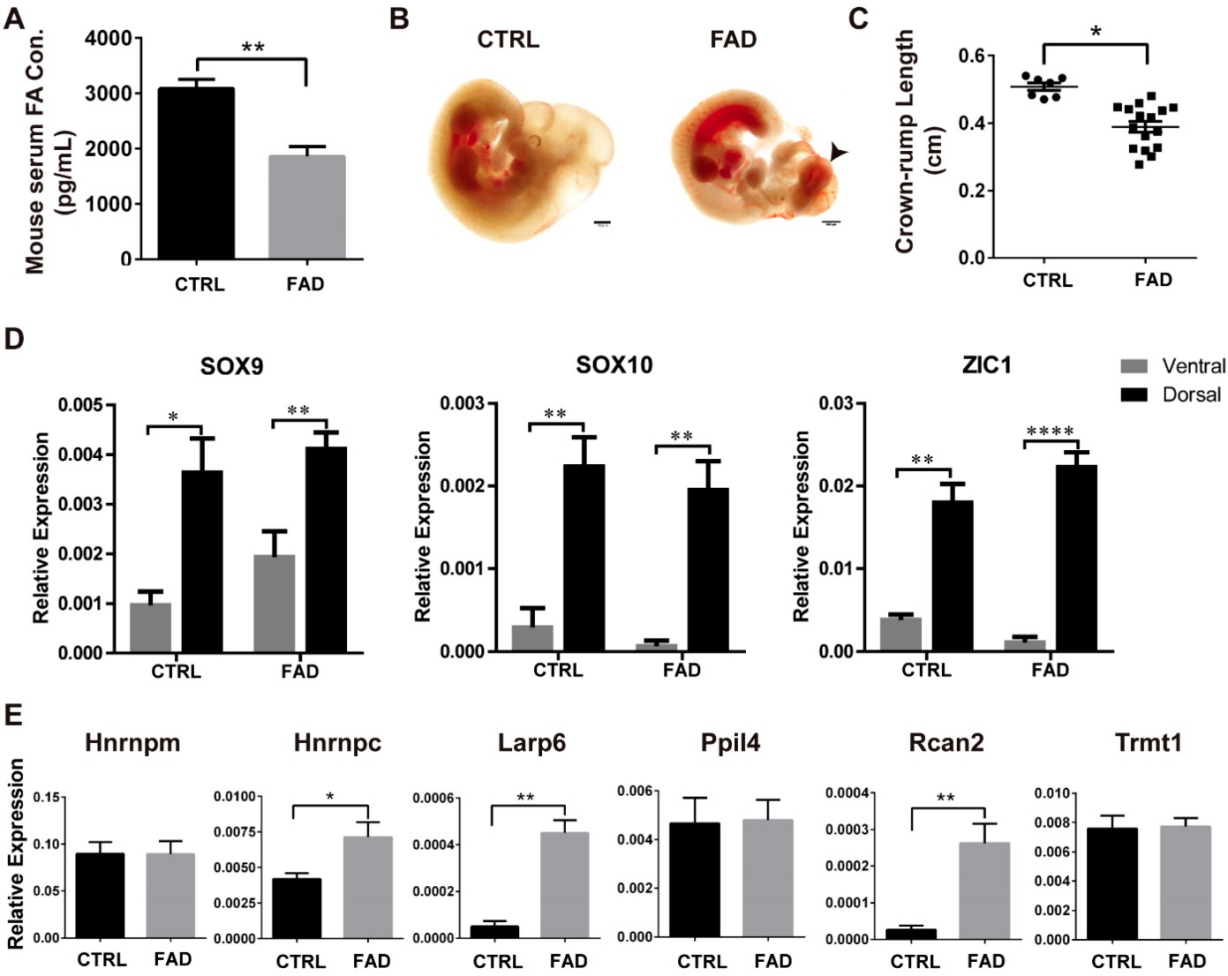
** Establishment of FAD mice models *in vivo* and RNA binding protein expressions verification in dorsal neural tube-portion of mice embryos. (A)** ELISA results showed that mice had reduced FA serum levels when having a FAD diet with the intestinal antibiotic succinyl sulfathiazole treatment for 12 weeks. (B) Bright field pictures showed that FAD led to abnormal neural tube development. FAD groups exhibited failure of neural tube closure (black arrow head). (C) Embryos isolated from dams fed with FAD diet had significantly shorter crown-rump lengths (CRL). (D) Higher expression of NCC-markers in the dorsal neural tube parts of mouse embryos from CTRL and FAD groups. (E) Verification of the expression of previously screened-out 6 RBPs in the FAD mouse model by real-time PCR analysis. * P<0.05, ** P<0.01, **** P<0.0001. Scale bars, 500 μm.

To investigate the effect of FA deprivation on the neural tube closure and neural crest development *in vivo*, C57BL/6J mice fed with FAD diet for 12 weeks were mated and the embryos were harvested at 10.5 dpc for investigation, as normal neural tubes close between 9.5 dpc and 10.5 dpc 47. The serum FA levels of control mice were 3000-4000 pg/ml; whereas the serum FA levels of FAD mice were approximately half of the control (p < 0.01) (Fig. 4A). The cranial neural folds fused properly in the normal control embryos at 10.5 dpc whereas the typical “cauliflower like” morphology of exencephaly was exhibited in FAD group (Figure 4B, black arrowhead). To quantitate the effects of FAD on the development of embryos, the NTD ratio and the crown-rump length (CRL) of 10.5 dpc *embryos* were compared between FAD group and control group. The FAD group embryos had a higher NTDs ratio compared to embryos from dams fed with the control diet (31.58% versus 3.1%, respectively; summarized in Table 2). The results of CRL measurement showed that FAD 10.5 dpc embryos were significantly smaller in size compared to the control 10.5 dpc embryos (Figure 4C).

To determine whether FAD influence the RBPs expression in neural crest-derived cells *in vivo*, we performed real-time PCR analysis to identify the mRNA level of NCC markers and RBPs in the dorsal neural tube part of 10.5 dpc mouse embryos in both the control (CTRL) group and the the FAD group. Real-time PCR analysis showed that the expression of NCC markers *Sox9*, *Sox10* and *Zic1* were significantly higher in dorsal neural tube part of mouse embryos than in the ventral counterparts, suggesting the enrichment of neural crest-derived cells in dorsal neural tube part of mouse embryos (Figure 4D). Furthermore, *Hnrnpc*, *Larp6* and *Rcan2* were found to be significantly up-regulated in the dorsal neural tube part of mouse embryos in the FAD-group compared to those in the CTRL group (Fig 4E). Collectively, all these findings suggest that embryos developing under folate-deplete conditions exhibited higher incidence of NTDs; meanwhile, expressions of *Hnrnpc*, *Larp6* and *Rcan2* in the neural crest-derived cells of 10.5 dpc mouse embryos were also significantly increased in FAD group* in vivo*, which to some extent in accordance with the results we observed *in vitro*.

### The Down-regulations of *LARP6*/*RCAN2*/*HNRNPC* under FAD Conditions are Accompanied with Consistent Increases in H9-NCC Viability

To further assess the relationship between RBPs expression and FAD-induced human NCC dysfunctions, *HNRNPC*, *RCAN2* and *LARP6* in H9-NCCs were depleted by shRNAs targeting individual gene respectively (shHNRNPC-group, shLARP6-group and shRCAN2-group) and their effects on cell viability and migration under FAD condition were investigated *in vitro*.

**Figure 5:**
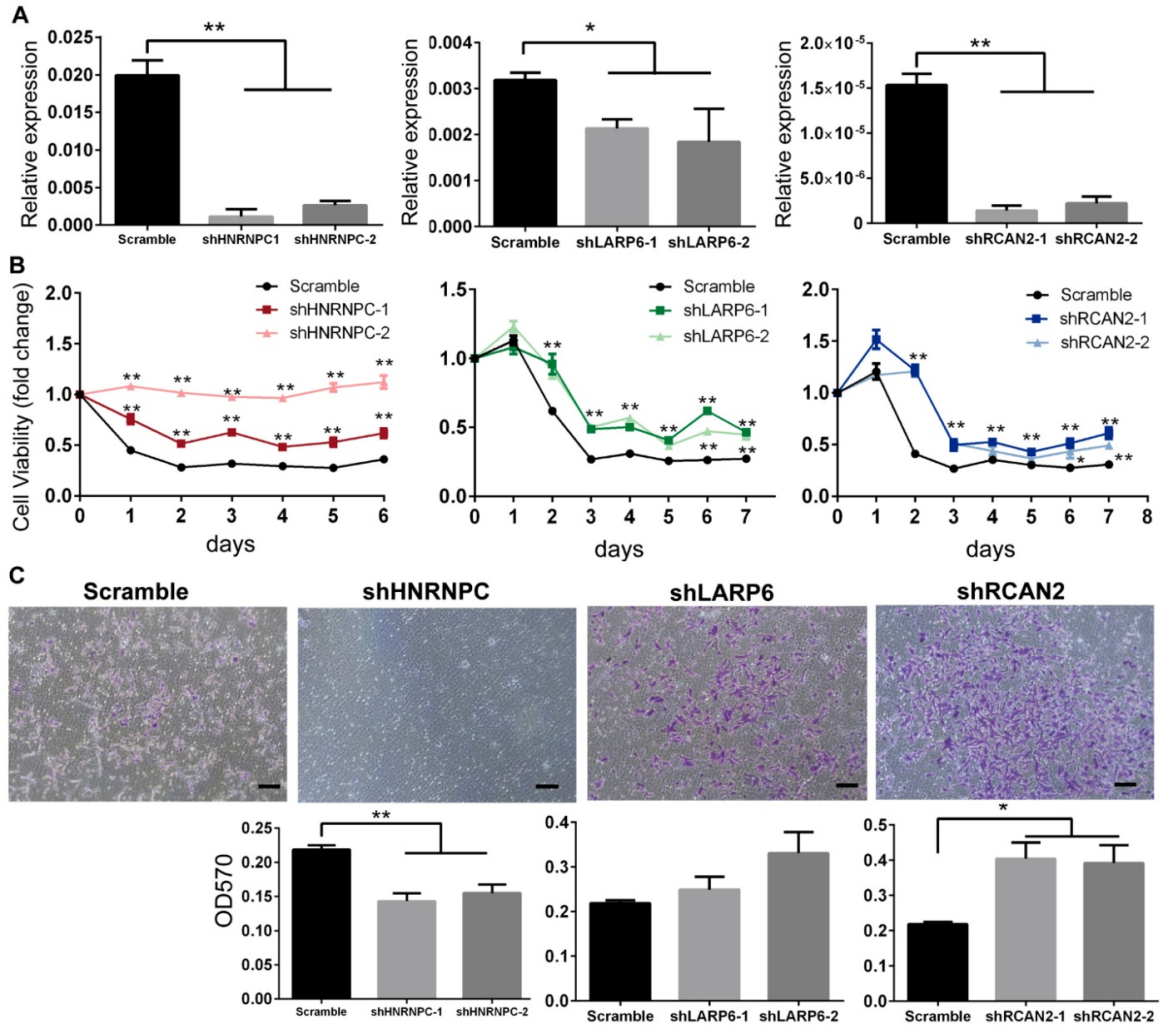
** Knockdown of *HnRNPC*,* LARP6* and *RCAN2* in FAD H9-NCCs *in vitro* model increased the viability of H9-NCCs.** (A) Real-time PCR verifications of shRNA knockdown of *hnRNPC*, *LARP6*, and *RCAN2*, respectively. (B) CCK-8 tests showed that knockdown of each RBP could promote NCC viability. (C) Transwell showed down-regulation of *hnRNPC* inhibited the H9-NCC migration under FAD culture, while down-regulation of* LARP6* did not show significant changes and knockdown of *RCAN2* promoted H9-NCC migration. * P<0.05, ** P<0.01. Scale bars, 100 μm.

As shown in Fig. 5A, real-time PCR results confirmed that the mRNA levels of RBPs in the shRNA-silenced H9-NCC groups were significantly lower than in the scramble control group. CCK8 assays showed that the H9-NCC viabilities were significantly increased after knockdown of each RBP in FAF culture (Fig. 5B). The number of transmigrated H9-NCCs in the shRCAN2-group increased significantly compared to the control H9-NCCs, while hnRNPC-knockdown H9-NCCs showed impaired migration ability; however, LARP6 depletion does not result in a significant variance in H9-NCC migration (Fig. 5C).

To further investigate that whether up-regulation of RBP genes could recapitulate FAD phenotype and lead to the dysfunction of NCCs, HNRNPC, RCAN2 and LARP6 in H9-NCCs were overexpressed and their effects on cell viability and migration under normal culture condition were investigated *in vitro*. As shown in Supplementary Fig. 2A, three RBP genes were successfully overexpressed in H9-NCCs and were confirmed by real-time PCR analysis. Overexpression of RCAN2 and HnRNPC did not alter the viabilities of H9-NCCs while overexpression of LARP6 significantly decreased the viabilities of H9-NCCs (Fig. S2B). C-MYC is a transcription factor plays an important role in cell proliferation, apoptosis and metabolism 48. In our study, we observed that the transcription of *C-MYC* was significantly down-regulated in LARP6-overexpressed H9-NCCs (Fig. S2C), suggesting that overexpression of LARP6 might inhibited the viabilities of H9-NCCs via C-MYC-mediated pathways. Nevertheless, overexpression of these three RBP genes did not affect the migration of H9-NCCs (Fig. S2D).

## Discussion

The NCCs has been a fascinating group of cells because of its multipotency, long range migration through embryo and its capacity to generate a prodigious number of differentiated cell types 49. However, the biological studies of human NCCs are extremely challenging due to the ethical issues. NCCs derived from hESCs offer an effective approach to study the human neural crest development and the pathology of related-diseases 50. RBPs play vital functions in a range of biological processes and are involved in many diseases 37, including multifactorial developmental anomalies. To investigate the roles of RBPs in FAD induced NCC dysfunction, we built three types of FA deficient H9-NCCs models *in vitro* and screened out the differentially expressed RBPs. Additionally, a FAD mouse model was used to verify the expression of RBPs in the dorsal neural tube parts of embryos enriching neural crest-derived cells and further narrowed down the differentially expressed RBPs into hnRNPC, LAPR6 and RCAN2. Furthermore, knocking down *hnRNPC* in FAD culture medium promoted H9-NCC viability but was negatively correlated with H9-NCC migration; while *LARP6*-knockdown H9-NCCs exhibited significantly increased cell viability without significant effects on H9-NCC migration. Knocking down *RCAN2* promoted H9-NCC viability as well as migration under FAD conditions. In addition, overexpression of LARP6 could partially mimic the effects of FAD by decreasing the viabilities of H9-NCCs cultured in normal condition. Our results provide insights into the RBPs function in FAD-induced dysfunction of NCCs.

Deriving H9-NCCs from H9-hESCs overcomes species variation and offers a platform for NCC developmental studies. In our work, three *in vitro* models of FAD H9-NCCs were established, and the results showed that FAD induced NCC apoptosis and impaired cell viability and migration. The FAF group mimics the insufficient folate-intake by culturing H9-NCCs in FA-depleted medium. Similar models have been reported in a few studies in which in FA depletion medium, the proliferation of Rhesus ESCs also decreases, and embryoid body (EB) formation and neuronal differentiation are affected 43. While the MTX-group simulates the interruption of FA metabolism using the antagonist MTX, it is reported that MTX dramatically affects the neural differentiation of the induced pluripotent stem cells (iPSCs) derived from spina bifida patients and FA exposure partially rescues this severe phenotype 29. The shRFC-group imitates the malabsorption of FA with the knockdown of main FA carrier RFC. RFC is the major route for the membrane transport of folates into systemic tissues at physiological pH 7.4 51. The *Rfc-*mutated mouse shows NTDs phenotypes and is rescued by FA 52. Accordingly, the low level of FA is observed in all three FAD H9-NCC models. Taken together, our results are consistent with other studies and suggest that the viability and migration of NCCs have a high demand for folates.

Dietary factors have long been thought to play an important role in determining susceptibility to NTDs and neurocristopathies; therefore, we adopted the well-established FAD diets to feed C57BL/6J mouse 44, 45, 53-55 and observed that FAD blocked the *de novo* initiation of neural tube closure at the forebrain-midbrain (FB-MB) boundary of mouse embryos with the growth retardation. It is reported that a mouse maternal FA deficiency results in marked impairment of eye development, which is very similar to ocular malformations caused by the lack of migration and differentiation of NCCs 55. Serine hydroxymethyltransferase 1 (*SHMT1*) deficient mice exhibit FA-responsive NTDs and impairments in *de novo* purine and thymidylate (dTMP) biosynthesis; mouse embryos from dams fed the FAD diets show a lower growth rate due to the failure of cell proliferation 44. The FAD mouse embryos in our study showed aberrant neural tube closure and shorter CRL; these anomalies are consistent with other related studies. However, the mechanism of how FAD diets effects neural tube development and NCC function, especially in the gene expression changes at the posttranscriptional level, are not fully understood.

RBPs, the proteins known and annotated as 'RNA-binding', are implicated in a spectrum of genetic diseases, and neurological disorders are the most prominent group of diseases caused by RBP mutations 37. RBPs can modulate the balance between self-renewal and differentiation of stem cells as well as cell migration capabilities in response to developmental or environmental cues, thus, influencing the formation of tissues and organs 56. Defects in RBPs might alter the level or the function of proteins involved in cell differentiation, cell division, integrity checkpoints, or cellular responses to stimuli, which are all processes in which accurate regulation is essential. The up-regulation of four RBPs in FAF H9-NCCs (HNRNPM, HNRNPC, LARP6, and TRMT1) and two RBPs (PPIL4 and RCAN2) in shRFC H9-NCCs were found through *in vitro* FAD H9-NCC model screening. Furthermore, the expression changes of hnRNPC, LAPR6 and RCAN2 in neural crest-derived cells were verified in FAD embryos and their effects on the viability and migration of FAF H9-NCCs were investigated. Our results expand the knowledge of expression patterns of RBPs in NCCs under FAD conditions.

HnRNPC belongs to the subfamily of ubiquitously expressed heterogeneous nuclear ribonucleoproteins (hnRNPs); this family of proteins functions in many aspects of mRNA metabolism and multiple cellular pathways 57, 58. There are approximately 20 hnRNPs named from A1 to U 59. The only member of hnRNPs family reported relevant with FAD induced NTDs is hnRNP-E1, which is up-regulated under FAD conditions and, in turn, stimulates folate receptor biosynthesis to ensure folate homeostasis 60, 61. HnRNPC is up-regulated in many tumors and silencing *hnRNPC* is reported to suppress the metastatic potential of glioblastoma, an aggressive type of brain tumor 62. In our study, *hnRNPC* depletion increased the viability of FAF H9-NCCs but decreased cell migration while overexpression of HnRNPC alone did not affect the viability and migration of H9-NCCs under normal culture condition. These findings suggested that HnRNPC is not the key player that directly correlated with the FAD-induced NCC dysfunction.

Our results further demonstrated that knockdown of LARP6 showed similar promoting functions on the viability of FAF H9-NCCs and overexpression of LARP6 could reduce the viability of H9-NCCs under normal culture condition, indicating that LARP6 might play an important role in the regulation of NCC viability and directly correlated with the FAD could impair the NCCs viability through LARP6-mediated pathways. These findings highlight a function of LARP6 that is distinct from its established role in regulating translation of collagen mRNAs 63-65.

Regulators of Calcineurin (RCAN) proteins regulate the calcium-activated phosphatase calcineurin. RCAN2 is linked to the nuclear factor of activated T cells (NFAT) signaling pathway in regulating muscle and bone development 66, 67, while the expression of RCAN2 negatively correlates with mesenchymal stem cells (MSCs) proliferation and cartilage differentiation 68. In our study, although overexpression of RCAN2 alone could not mimic the FAD condition and did not affect viability and migration of H9-NCCs under normal culture condition, knockdown of *RCAN2* to some extent rescued the impaired viability and migration of FAF H9-NCCs under FAD condition. These findings suggesting that RCAN2 might function as an important collaborator that involved in the FAD-induced NCCs dysfunction.

In summary, we built three models of H9-NCCs under FAD conditions, and we proved that FAD negatively affected the NCC viability and migration abilities and promoted human NCC apoptosis *in vitro*, and the occurrence of NTD in mouse embryos *in vivo*. In addition, for the first time, we showed that three RBPs, namely, *hnRNPC*, *LARP6* and* RCAN2,* were up-regulated both in the H9-NCC FAD models *in vitro* and in the mouse FAD model *in vivo*. It is worth noticing that knocking down of *LARP6* under FAD culture medium promoted H9-NCC viability while overexpression of the *LARP6* could partially mimic FAD effects and led to significantly decrease in the H9-NCCs viability. Taken together, our results demonstrate an important regulatory mechanism for RBPs underlying the FAD-related abnormalities in NCC development.

## Supplementary Material

Supplementary figures and tables.

## Figures and Tables

**Table 1:** RBPs that are significantly differently expressed in the GEO dataset No. GSE3548.

CSDE1	DAZ2	DAZ2	DAZL	THUMPD1
SNRPN	MYEF2	RCAN2	TRMT1	TRMT2A
FMR1	PPIL4	RBM47	HNRNPM	HNRNPA0
HNRNPC	PNO1	CHERP	MEX3C	LARP6
LARP7	TLR2	ESRP1	SYNCRIP	TUT1
PCBP3	FUS	PABPC5	KIN	YTHDC1
RAVER2	PARP14	PPARGC1A	ZNF74	U2AF2

**Table 2:** The NTDs and reabsorption rates of each group.

Group	Female litters (n)	Total embryos (n)	Viable Embryos (n)	Reabsorption n (%)	Normal embryos n (%)	NTD ratio n (%)
12w-FAD	5	39	38	1 (2.5%)	26 (65%)	12 (31.58%)
12w-Ctrl	4	35	32	3 (9.68%)	31 (88.57%)	1(3.13%)
