# Supplementary material for: Up-regulation of RNA Binding Proteins Contributes to Folate Deficiency-Induced Neural Crest Cells Dysfunction
Source: Int J Biol Sci. 2020 Jan 1;16(1):85–98. doi: 10.7150/ijbs.33976 (PMC6930370; doi:10.7150/ijbs.33976)
Supplement: Supplementary file 1 — Supplementary figures and tables. [file ijbsv16p0085s1.pdf]

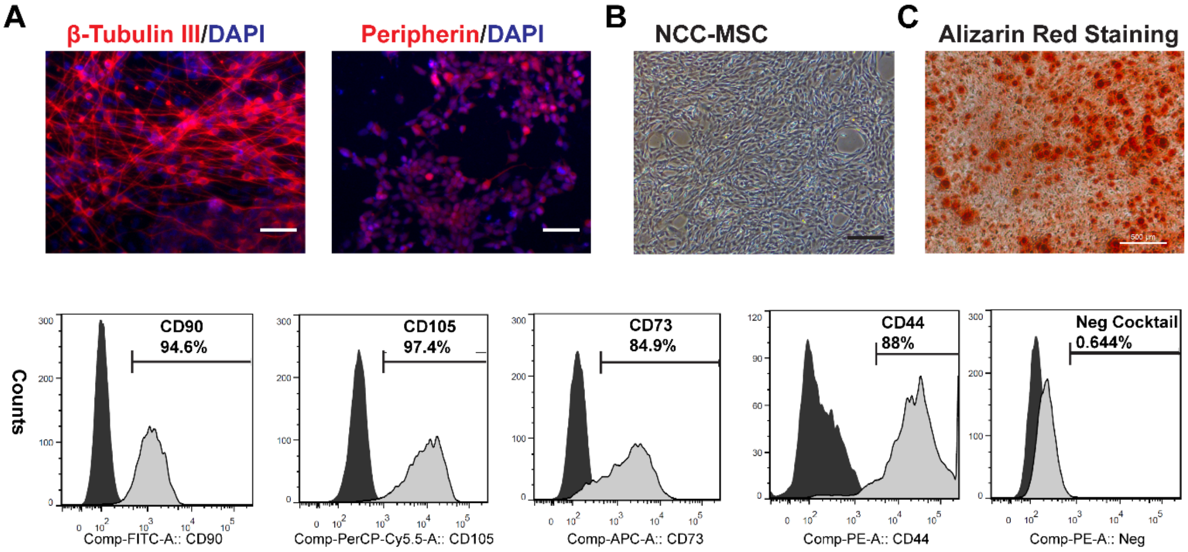

Supplementary Figure 1. Differentiation of H9-NCCs into peripheral neuron and mesenchymal stem cells. (A). Immunofluorescence staining of H9-NCC-differentiated neurons with peripheral neuron markers β-Tubulin III and Peripherin. (B) Morphology of mesenchymal stem cells (MSCs) differentiated from H9-NCC. (C) Alizarin Red staining of H9-NCCs-differentiated MSCs after cultured in osteogenic induction medium for 14 days. (D) Flow cytometry analysis of H9-NCCs-differentiated MSCs for expression of MSCs surfaces markers.

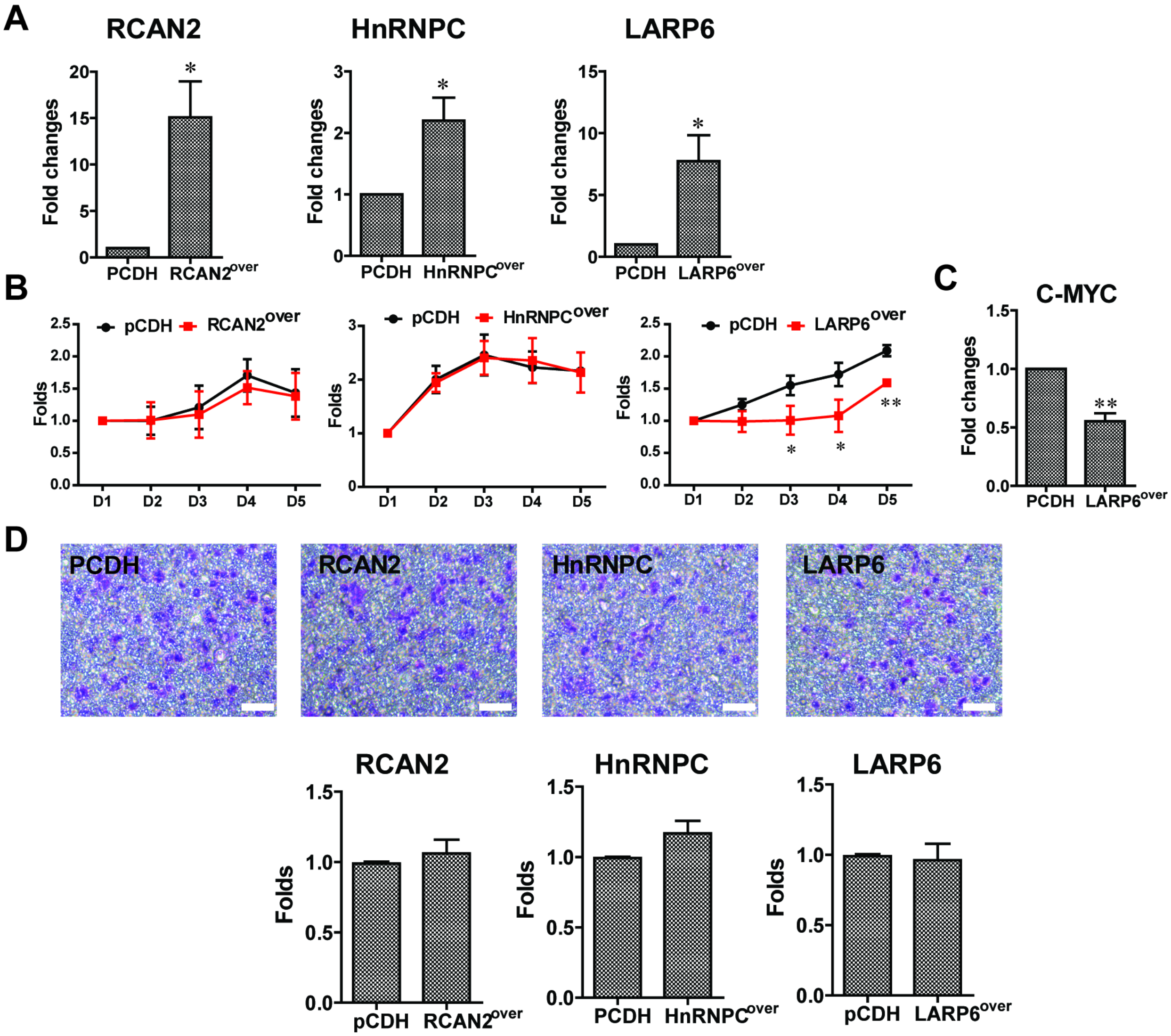

Supplementary Figure 2. Overexpression of LARP6 in H9-NCCs cultured in normal culture condition decreased the viability of H9-NCCs. (A) Real-time PCR verifications of overexpression of HnRNPC, LARP6, and RCAN2, respectively. (B) CCK-8 assay results showed that overexpression of LARP6 decreased the H9-NCC viability. (C) Expression of C-MYC was down-regulated in LARP6-overexpressed H9-NCCs. (D) Transwell showed that overexpression of three RBP genes did not affect the migratory abilities of H9-NCCs. * P<0.05, ** P<0.01. Scale bars, 100 μm.

Supplementary Table S1. Sequences of shRNA used in gene knockdown assays

**shRNA sequence to knock down RFC**	
**Gene Names**	**Sequences (5'-3')**
shRFC1	TTCACATTCTGAACACCGTCG
shRFC2	TATAACTGGAACTGCTTGCGG
**shRNAs Sequences to knock down RBPs in NCCs**	
**Names**	**Sequences (5'-3')**
shRNA-LARP6	1. GCACATGCTTTGAAGTATTCA
	2. GCAAGATGCTCCTGGTCTATG
shRNA-HNRNPC	1. GAGATGTACGGGTCAGTAACA
	2. GGACCTACTGGATGATGATGA
shRNA-RCAN2	1. GGGACTGTTTCGGACTTATGA
	2. GCTCTACTTTGCACAGGTTCA

Supplementary Table S2. Sequences of primers used in real-time PCR analysis

**Primers for human ESCs and NCCs markers**	
**Gene Names**	**Sequences (5’-3’)**
OCT4	F: CCCCTGGTGCCGTGAA
	R: GCAAATTGCTCGAGTTCTTTCTG
NANOG	F: ATGCCTCACACGGAGACTGT
	R: AAGTGGGTTGTTTGCCTTTG
SOX2	F: TTGCTGCCTCTTTAAGACTAGGA
	R: CTGGGGCTCAAACTTCTCTC
HNK1	F: GAAAGCAGCCTCCTTCGAGAAC
	R: CCTCATTCACCAGCACTGGCTT
PAX3	F: AGAAGGCCAAACACAGCATC
	R: TTCTGCGCTGTTTCCTCTTT
SOX9	F: CCCCAACAGATCGCCTACAG
	R: TTCTGGTGGTCGGTGTAGTC
SOX10	F: CACCTGCACAACGCTGAG
	R: CTTTCTTGTGCTGCATACGG
ZIC1	F: GCATCCCAGTTCGCTGCGCAAA
	R: GGAGACACGATGGTGGGAGGCG
AP-2α	F: ATTGACCTACAGTGCCCAGC
	R: ATGCTTTGGAAATTGACGGA
P75	F: GTATTCCGACGAGGCCAACC
	R: GTGTAATCCAACGGCCAGG
**Primers for folate transporters**	
**Names**	**Sequences (5’-3’)**
RFC	F: GCTCCTACCAGTTCCTCGTG
	R: GGCAAAGAACGTGTTGACCC
FOLR	F: GACCACAGCTCTTTCTTCAGGGA
	R: TGTTGGTAGAACAGCAGGCAT
PCFT	F: TCACTCTACCCAGCCACTCT
	R: GACCTCCAGTTGCTTGGTGT

**Primers for RBPs in mouse embryos**	
**Gene Names**	**Sequences (5’-3’)**
Hnrnpm	F: TTGCTGTTTGATAGACCGAT
	R: TAATCCCATGCCAATACCAC
Hnrnpc	F: ATCCTCGGTCCATGAATTCCC
	R: ATCCTCGCCAGCTACAGCA
Trmt1	F: CTCCTTCATGCTGGTTTCCGAGT
	R TTCAGACAGCCGCTCCCGTTT
Larp6	F: GCCATTGTGGAGTTCGAGGA
	R: AGGGATCTGCTTAGGTGGGT
Ppil4	F: GATTTCAGCCAGTCTGTTGC
	R: CAAATTGGCTGGTTTATCCTGT
Rcan2	F: TACATGCTATTGCGGTTCGT
	R: TTTCTTGGATTTAGCGTCACC
**Primers for RBPs in H9-NCCs**	
RCAN2	F: GACTGGGCTGTCACTCGTTG
	R: TAGCTGGAACGTCACACAGTC
HNRNPC	F: AACCGAGGAAAAGCAGGTGT
	R: CTGACGTTTCGAGGGCACTA
LARP6	F: TCAGTGCGGATCCTCAAACC
	R: TTTGATGGCTGCTTCCACCT
HNRNPM	F: GCCCTTTGGTGGTGGTATG
	R: TCCCTGCTTTGCAATGATCT
TRMT1	F: CTCCCACGCCTGTAAGAAC
	R: ACACTGAGAATGCGGAACG
